# Microalgae-based integrated treatment of tannery wastewater: emphasizing microbial synergy for sustainable remediation

**DOI:** 10.1039/d6ra01394e

**Published:** 2026-07-29

**Authors:** Ricky Rajamanickam, Roshna Parveen G., Pollyanna Vanessa dos Santos Lins, Hugo Juarez Vieira Pereira, Dison S. P. Franco, Jordana Georgin, Lucas Meili, Rangabhashiyam Selvasembian

**Affiliations:** a Department of Biology and Environmental Science, Centre for Ecology and Evolution in Microbial Model Systems (EEMiS), Linnaeus University 39231 Kalmar Sweden; b Department of Environmental Science and Engineering, School of Engineering and Sciences, SRM University-AP Amaravati Andhra Pradesh 522240 India rambhashiyam@gmail.com; c Laboratory of Processes Applied to Water Treatment, Center of Technology, Federal University of Alagoas Maceió Alagoas Brazil lucas.meili@ctec.ufal.br; d Department of Civil and Environmental. Universidad de la Costa, CUC Barranquilla Atlántico Colombia; e Institute of Chemistry and Biotechnology, Federal University of Alagoas Maceió Alagoas Brazil; f Centre for Interdisciplinary Research, SRM University-AP Amaravati Andhra Pradesh 522240 India

## Abstract

Tannery wastewater (TWW) presents a complex and toxic mixture of pollutants, including heavy metals such as chromium, cadmium, lead, and nickel, as well as organic compounds, surfactants, sulfides, chlorides, and dyes. Conventional treatment methods often lead to high operational costs, sludge generation, and disposal issues. This review critically examines the integration of microalgae-based systems with physical, chemical, and biological methods for advanced TWW remediation, emphasising mechanistic insights and consortia-driven treatment. Microalgae facilitate pollutant removal through biosorption, bioaccumulation, enzymatic transformation, and the mitigation of oxidative stress. Specific enzymatic mechanisms, such as chromate reductase-mediated Cr(vi) reduction and peroxidase-driven degradation of phenolics, enable precise detoxification at the cellular level. In multi-species consortia, synergistic interactions optimise nutrient uptake, broaden substrate specificity, and enhance EPS-mediated heavy metal immobilisation. This review delineates how microalgae–bacteria, microalgae–fungi, and microalgae–microalgae systems contribute distinct advantages *via* mutualistic nutrient exchange, quorum sensing regulation, and biofilm resilience under high pollutant stress.

## Abstract 2

Microalgae-based consortia enhance tannery wastewater treatment through biosorption, bioaccumulation, enzymatic detoxification, and EPS-mediated heavy metal immobilisation, enabling efficient pollutant removal and sustainable resource recovery.

## Graphical abstract

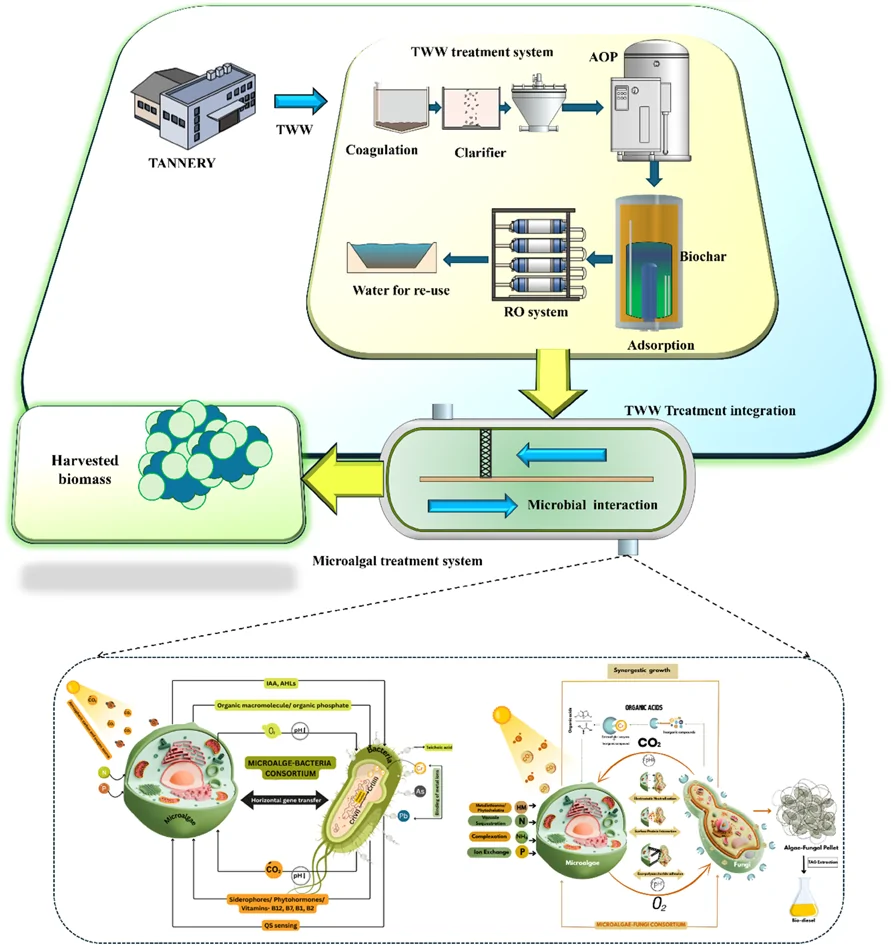

## 1. Introduction

The tanning industry plays an important role in the global economy, showing consistent growth in recent years.^1^ The tanning process is carried out in a biological environment with an abundance of organic and inorganic substances. High levels of total dissolved solids (TDS), biochemical demand for oxygen (BOD), phosphate (P), nitrogen (N) and heavy metals (HMs) are found in tannery wastewater (TWW).^2,3^ If untreated TWW is released directly into the environment, it poses major threats to the ecosystem, as it reduces the infiltration of sunlight into aquatic environments, thereby reducing photosynthetic activity and creating anaerobic conditions. On the Earth's surface, it alters the structure of the microbial community and reduces its activity.^4,5^ Similarly, if the toxic components of TWW reach the food chain, they pose a host of health problems to all the living organisms.^6^

TWW needs to be adequately treated to reduce pollution parameters within allowable limits, thereby mitigating environmental hazards. Sedimentation, coagulation, flotation and adsorption are some of the traditional procedures used to remediate tannery effluents.^7^ Biological technologies, such as activated sludge, constructed wetlands, and fluidised bed reactors, can be used aerobically or anaerobically in addition to these traditional techniques.^8,9^ State-of-the-art methods for the effective treatment of TWW are being developed, including membrane separation, enhanced oxidation, catalytic heat treatment, and electrochemical treatment.^10,11^

To properly treat TWW and generate value-added goods, cost-effective and environmentally friendly treatment techniques must be developed. In this sense, microalgae seem to be helpful for the treatment of tanneries and other industrial effluents.^12,13^ Numerous benefits differentiate microalgae-based remediation from other remediation techniques, making it a more popular and long-lasting restoration solution.^14,15^ To create value-added biomass that can be used in the manufacture of various products, microalgae use various organic and inorganic compounds from TWW as a source of nutrients.^16^ Ion exchange techniques, adsorption, precipitation of heavy metals using microalgae, or covalent bonding are the ways in which heavy metals are accumulated.^13^

Microalgae-based treatment can facilitate CO_2_ sequestration through the process of photosynthesis. When wastewater is exposed to the sun's UV-B and UV-A rays, it can effectively eradicate pathogens such as *E. coli*.^17^ However, establishing setups and reactors for growing microalgae using wastewater presents some challenges. The first is the need for a large amount of land; the second is that the conditions of cultivation in wastewater, such as pH, turbidity, colour and composition, impact the growth of microalgae and, consequently, the production of biomass. Thirdly, the production of microalgae and biomass, as well as the efficiency of treatment, are also affected by environmental factors.^16^

The chemical demand of oxygen (COD), total phosphorus (TP), and total nitrogen (TN) decreased by 56.7%, 97.6%, and 71.7%, respectively, in the intercropping of microalgae with *Tetraselmis* sp.^1^ There are more than 3000 types of photosynthetic aquatic microalgae, and they reproduce rapidly. Microalgae are an efficient way to remediate wastewater while simultaneously creating biomass that can be used to make a variety of products with added value.^18–20^ The researchers found that after 20 days of treating tannery effluent with *Chlorella* sp., HMs were significantly excluded, and 60% of the CO_2_ was consumed.^21^ The main components of microalgae cells are carbohydrates, lipids, and proteins. By dissolving its cellular structure, the lipid can be removed and used for oil recovery through the process of transesterification to produce biofuel.^22,23^ The carbohydrate can be digested and fermented anaerobically to provide butanol or ethanol.^24^*Chlorella vulgaris* was used by another team of researchers to produce bioethanol.^19^ Pigments found in microalgae cells, including β-carotene, chlorophyll, astaxanthin, phycobiliproteins, and xanthophylls, can be used to make cosmetics, nutraceuticals, and bio pigments.^25^ In addition, through various combinations with microbial fuel cells, microalgae can potentially be utilised in the commercial production of hydrogen and energy in industry.^26,27^

Consequently, basic bioremediation is successful but unsatisfactory, generating many secondary contaminants. Microalgae remediation, on the other hand, recovers diverse components for the creation of various value-added products, while also utilising the wastewater pollution load as a nutrient source. The potential and challenges that may arise from the application of research, considering all the foreseen possibilities, were examined in this study. Additionally, the ability of microalgae to remove secondary pollution in a sustainable manner is highlighted. The mechanics of nutrient absorption and the difficulties it presents are covered in detail.

This review focuses on the integration of biomass recovery and microalgal systems for the treatment of TWW and the production of various value-added products. It explores the synergistic relationship between biomass production and key influencing factors, mechanisms, and the toxicity of tannery effluent, along with its impact on microalgal cultivation. The classification and characteristics of microalgae are discussed in detail, alongside their applications in treating tannery effluent and the role of different operational parameters in determining treatment efficiency. Particular emphasis is placed on integrated cultivation systems such as microalgae–bacteria, microalgae–fungi, and microalgae–microalgae consortia, which enhance treatment performance through mutual nutrient exchange, quorum sensing, and improved biofilm resilience under high pollutant stress.

## 2. Microalgal potential in the removal of pollutants from tannery wastewater

**Fig. 1:**
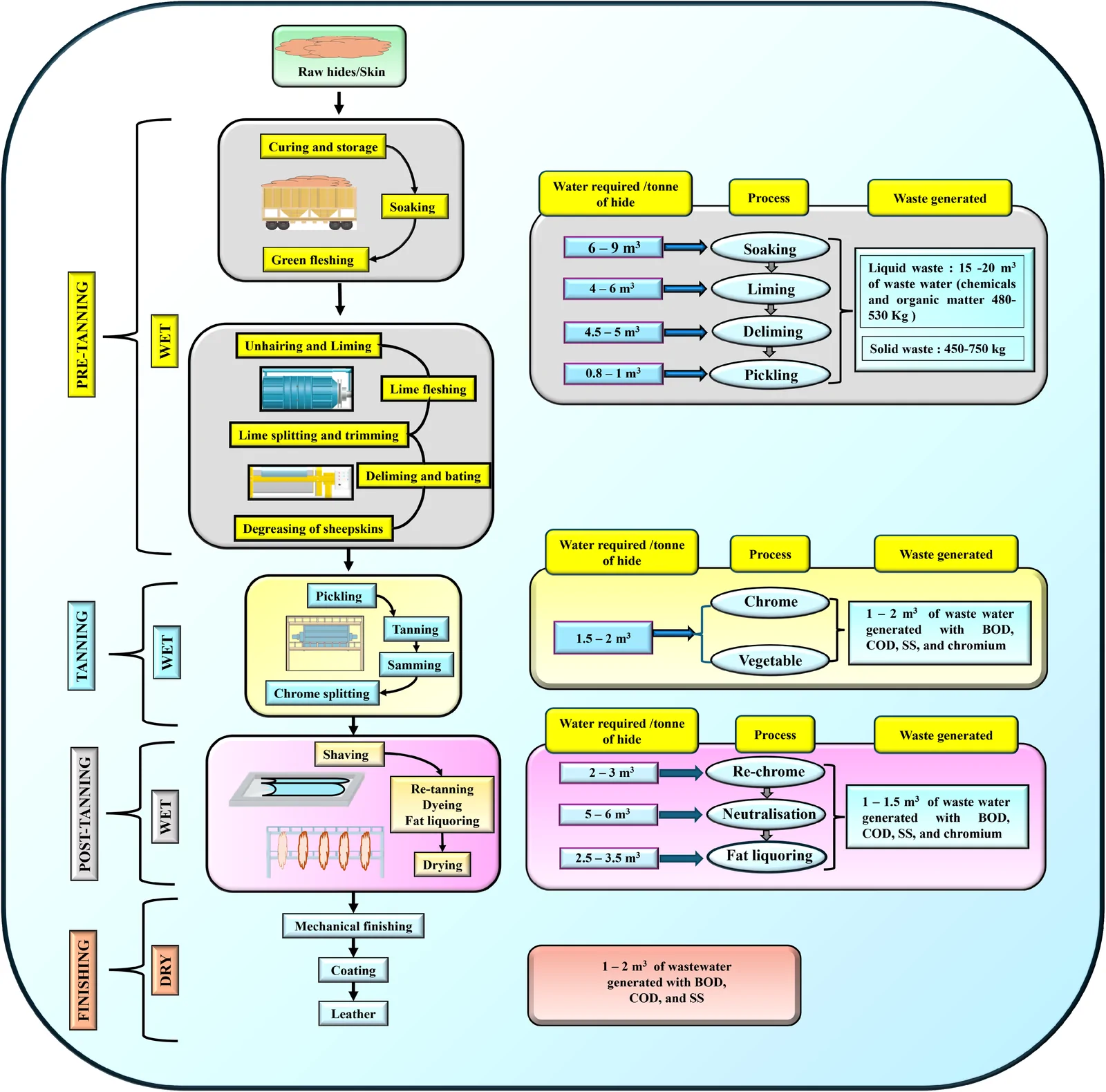
Tannery wastewater generated in each stage of the tannery process.

TWW is one of the most complex and heavily polluted industrial effluents, generated from multiple stages of leather processing, including soaking, liming, tanning, dyeing, and finishing. Fig. 1 presents the generation of wastewater in each stage of the tannery process.^28^ This effluent is characterised by a high load of organic and inorganic pollutants, such as sulfides, ammonia, chlorides, surfactants, and heavy metals like chromium (Cr), lead (Pb), cadmium (Cd), and zinc (Zn), along with high values of COD, BOD, total suspended solids (TSS), and TDS. The presence of persistent organic pollutants (POPs) like phthalates and polycyclic aromatic hydrocarbons (PAHs) further exacerbates their toxicity and environmental persistence.^3^ The concentration of these contaminants varies widely depending on the raw materials, chemicals used, and processing techniques employed. Given their toxicity, bioaccumulative nature, and resistance to conventional treatment processes, a detailed understanding of the physicochemical characteristics of TWW is essential for developing efficient and sustainable treatment strategies. This section compiles the reported concentrations of key pollutants in TWW and highlights their environmental and health implications, with a focus on the potential of microalgae-based bioremediation as an innovative and eco-friendly approach and the need for integration of treatment.

### 2.1 Heavy metals

Trivalent chromium (Cr III) is one of the most used heavy metals in the tannery industry, for leather dyeing due to its ability to stabilize collagen fibres and increase the strength of the product. Up to 40% of the chromium spent in industrial processes can end up in the effluent, often exceeding regulatory limits for environmental disposal. Recent studies have highlighted the persistent presence of Cr(iii) in tannery effluents, with concentrations ranging from 12.4 to 46 mg L^−1^, which exceed acceptable parameters for safe disposal. This raise concerns as to its potential conversion to the more toxic Cr(vi) under oxidative conditions. Chromium must be removed from wastewater to prevent water pollution. Different nations have implemented legislation requiring discharges of less than 0.5 mg L^−1^ of Cr.^8,29,30^ Other frequently detected metals in TWW include Zn, Ni, Cd, and Pb. These metals are harmful, bioaccumulative, and non-biodegradable, which increases their persistence in the environment. Continuous contact with these metals can pose significant health risks, including neurological damage, cardiovascular disorders, kidney complications, and an increased risk of cancer and diabetes.^31,32^ The use of microalgae-based treatment systems for removing heavy metals is both efficient and sustainable.

The following article provides examples of approaches and possibilities within this field.^33^ evaluated the ability of microalgae to remove heavy metals (Cd^2+^, Cr^3+^ and Pb^2+^) and produce high quality lipids. The addition of glycine betaine (GB) significantly increased the efficiency of heavy metal removal and lipid productivity. The best results were observed with Cr^3+^, where lipid productivity reached 101 mg per L per day and removal efficiency reached 85%. The industrialisation process led to the generation of increased amounts of toxic heavy metals. Yokwana *et al.* (2025) developed new bio-nanostructured adsorbents using *Haematococcus lacustris* microalgae cells mixed with graphene oxide-activated carbon (GO-AC) and graphene nanoplatelet-activated carbon (GNPs-AC). The GO-AC@Cyst-C nanohybrid showed the highest lead removal efficiency (more than 98%) due to its high specific surface area, oxygen and nitrogen-based functional groups, and favorable pore structure. This adsorbent also achieved the highest monolayer adsorption capacity of 25.58 mg g^−1^ according to the Langmuir model.^34^

According to the study by Tian *et al.* (2025), microalgae-derived magnetic biochar (MMBC) was prepared with a mixture ratio of algae powder to K_2_FeO_4_ of 1 : 4 at 200 °C (MMBC2001:4) and demonstrated excellent adsorption capacity, with optimized performance at pH ≤ 5.0. Adsorption in multimetallic solutions was slightly lower for individual metals due to competition but showed no significant reduction in overall capacity. The adsorption kinetics followed a pseudo-first-order model for single metals and a pseudo-second-order model for multimetallic solutions, while the adsorption isotherms were best described by the Langmuir and Freundlich models.^35^ According to a study by Wang *et al.* (2024), a coupled nanoscale zero-valent iron (nZVI) and microalgae system has been developed for treating copper-containing wastewater. The optimal concentration of nZVI (50 mg L^−1^, 50 nm) significantly enhanced microalgae biomass and stimulated the secretion of extracellular polymeric substances, thereby increasing the number of binding sites and functional groups on the microalgae surfaces. The system achieved a copper removal rate of 94.99% for wastewater containing 10 mg L^−1^ of copper, with removal facilitated by adsorption, absorption by organisms, and nZVI contribution.^28^

### 2.2 Organic pollutants

Some of the hazardous substances in tannery effluents include aromatic hydrocarbons, fatty acids, phenols, and phthalates, which are added at different stages of the process. The high COD and BOD values associated with these compounds underscore their polluting capacity and the challenge of effective treatment.^6,36,37^ The most sensitive organic pollutants are phthalates, which contaminate water bodies and disrupt the endocrine systems of aquatic organisms. Even after conventional processes, residues of these pollutants persist, raising concerns about environmental quality.^38^

The following studies highlight the remarkable potential of microalgae-based systems for removing organic pollutants while integrated into renewable energy generation. In a study by Ghanbarzadeh *et al.* (2022), the microalgae *Nostoc calcicola* ISC 89 demonstrated significant tolerance to phenanthrene, a polycyclic aromatic hydrocarbon (PAH), highlighting its potential for the remediation of PAH-contaminated environments. *N. calcicola* ISC 89 also exhibited increased antioxidant enzyme activity, lipid accumulation, and phenolic acid content.^39^ In a study by Rios Ramirez *et al.* (2024), marine microalgae grown in an airlift photobioreactor demonstrated significant efficiency in removing polycyclic aromatic hydrocarbons (PAHs) (up to 90%) and fixing CO_2_ (up to 0.20 g per L per day), producing lipid-rich biomass. Nitrates were added to the culture medium, and increased CO_2_ fixation.^40^

### 2.3 Microorganisms

Tannery waste creates unique conditions that favour the growth of microbial communities, which play essential roles in the decomposition of organic compounds and the transformation of pollutants. Local microorganisms often develop resistance to toxic substances, such as Cr(vi), which allows them to biotransform it into the less toxic Cr(iii). This conversion process involves adsorption and reduction and has been widely studied for bioremediation treatments.^41,42^ In addition, biological treatment systems for tannery effluent have demonstrated a very successful removal of COD, nitrogen and phosphorus. Microbial consortia such as BM-S-1 also play a critical role in reducing energy use and overall wastewater toxicity.^43,44^

### 2.4 Surfactants

Surfactants are used in tannery processes to remove fats and properly prepare hides for the following tanning and dyeing phases. These substances, classified as anionic, cationic, non-ionic, and amphoteric surfactants, are not biodegradable, making them stable in wastewater, increasing their persistence and toxicity. Surfactants impact the resilience of aquatic ecosystems to environmental stress, restricting their ability to reproduce and develop.^45^ The presence of surfactants causes eutrophication due to the increased solubility of contaminants and reduces the efficiency of biological treatments by inhibiting bacterial activity.^46,47^

Surfactants have been shown to form organometallic complexes with HMs, thereby increasing environmental toxicity and causing difficulties to remediation processes.^48^ Advanced degradation procedures, such as chemical oxidation and bioremediation using specialized microorganisms, have shown potential in breaking down surfactants. Fungi and microalgae, including *Scenedesmus* sp. and *Aspergillus tubingensis*, are notably useful for biodegrading surfactants, decreasing their toxicity and improving the quality of treated effluents.^49–51^

### 2.5 Sulfides

Sulfides occur in TWW due to the use of sulfur compounds in steps such as waxing and degreasing. In addition to causing unpleasant odors, sulfides can release hydrogen sulfide gas (H_2_S) under anaerobic conditions, a highly toxic and corrosive gas.^52^ Sulfides can cause significant ecological effects at high concentrations, creating anoxic conditions in water bodies, reducing aquatic biodiversity. Treatment processes have been used to reduce sulfide levels in effluents called chemical precipitation by using iron compounds to produce insoluble iron sulfide or biological oxidation with sulfur-oxidizing microorganisms.^53^

López-Hernández *et al.* (2022) focused on the effectiveness of effluent recirculation in stabilization ponds with native microalgae as a tactic to reduce H_2_S releases. The method showed important improvements in wastewater treatment parameters, such as BOD (20.8% reduction), TSS (22.17% reduction), oils and fats (29.5% reduction), fecal coliforms (91.4% reduction), and H_2_S (48.9% reduction), avoiding odors and health risks.^54^ The research by Angelov *et al.* (2023) demonstrates the coupling of a two-compartment bio electrochemical system (BES) that joins bio methanation of ethanol stillage in the anodic section with oxygenated photosynthesis in the cathode compartment.^55^ This system performed substantial removals of COD (71.2–89.5%), ammonium (up to 76.5%), and total eradication of H_2_S in MEC mode, emphasizing its applicability for effective wastewater treatment and bioenergy generation. The following study explores a novel BES that combines oxygenated and anoxygenic photosynthesis for the upgrade of biogas composition by removing H_2_S and CO_2_ from the anode section with the simultaneous production of supplemental energy. The system did complete H_2_S removal and significant CO_2_ decline (81–85%), resulting in 94.1–96.2% methane content in biogas, showing its potential in effective biogas purification and energy upgrade.^55^

### 2.6 Ammonia

Nitrogen compounds used in tanning processes for degradation and neutralization of hides make ammonia a predominant constituent in TWW. Ammonia can exist as free ammonia (NH_3_) or as an ammonium ion (NH_4_^+^) in water, depending on the pH and temperature. Free ammonia is highly toxic to aquatic organisms, such as fish, and other sensitive water bodies.^56,57^ Additionally, NH_3_ is a source of eutrophication in water bodies, leading to algae overgrowth and a decrease in dissolved oxygen content.^58^ Ammonia can be oxidised into nitrate by biological processes comprising nitrification, which is performed by nitrifying bacteria such as *Nitrosomonas* and *Nitrospira*. Sarkheil *et al.* (2022) proposed the use of microalgae beads immobilized with sodium alginate from *Scenedesmus* spp. and *Chlorella* spp. as biosorbents for wastewater treatment in recirculating aquaculture systems (RAS). The algae granules demonstrated better removal efficiencies, with 42.85% for total ammonia nitrogen (TAN) and 44.90% for total phosphorus (TP), when compared to granules made only of alginate.^59^

### 2.7 Chlorides

Chlorides are introduced into tannery effluents through the application of common salt (sodium chloride) during the preservation of the leather before tanning. Excessive chloride levels increase the salinity of the effluent, reducing its quality and limiting its reuse or disposal into water bodies. High levels of chlorides are also dangerous for agriculture because they disrupt soil structure and nutrient balance.^60^ Typically, chloride removal is accomplished using desalination methods such as reverse osmosis or distillation. According to the research of Aregu *et al.* (2018), salt recycling during preservation can reduce chloride levels in effluents and significantly reduce operating costs.^61^ To facilitate a comparative identification of the chemical burden associated with leather processing, the following data presents a consolidated overview of the primary pollutants found in tannery effluents. Table 1 summarizes the main pollutants, their industrial sources, conventional concentration ranges, and the individual potential of microalgal species for their remediation, presenting a comprehensive baseline for assessing treatment needs.

**Table 1:** Major pollutants in tannery wastewater (TWW): sources, risks, treatment, and reported concentrations

Pollutants	Source in TWW	Concentration range (mg L^−1^)	Health/Environmental risks	Microalgae-based treatment	References
Chromium (Cr III/VI)	Leather tanning/dyeing	12–78 (total Cr), 12.4–46 (Cr III)	Cr VI: highly toxic, mutagenic; Cr bioaccumulates	Cr^3+^ removal: 85.8% with glycine betaine; lipid productivity: 100.98 mg per L per day	33, 62 and 63
Lead, cadmium, zinc	Dyes and process chemicals	Pb: 0.03–4.5; Cd: 0.005–0.1; Zn: 0.02–3.5	Bioaccumulative; neurotoxic; nephrotoxic	>98% Pb removal using GO-AC@Cyst-C multimetal removal with MMBC2001:4	34, 35, 62 and 64
Phthalates, PAHs	Fat liquoring, degreasing agents	PAHs: up to 1.5; phthalates: 0.1–12	Endocrine disruptors; persistent organic pollutants	Up to 90% PAH removal and 0.20 g per L per day CO_2_ fixation	38–40 and 65
Surfactants	Cleaning and preparation phases	Up to 3200	Not biodegradable; inhibit microbial treatment; eutrophication	Biodegradation by *Scenedesmus* sp., *Aspergillus tubingensis*	49–51, 65 and 66
Sulfides	Waxing, degreasing	5–800	Anaerobic gas emission; aquatic anoxia; toxicity	48.9% reduction by native microalgae	65 and 67–69
Ammonia (NH_3_/NH_4_^+^)	Neutralization and liming	3.20–21.38	Toxic to fish; causes eutrophication	42.85% ammonia, 44.9% phosphorus removal with *Scenedesmus/Chlorella* alginate beads	59 and 65
Chlorides (Cl^−^)	Leather preservation (salting)	715–20 490	Increases salinity; affects reuse and soil quality	Salt recycling strategy reduced Cl^−^ and operating cost	61 and 65
Phenols	Tanning and degreasing	0.4–10	Toxic; endocrine disrupting; resistant to degradation	Removal by algae such as *Chlorella vulgaris*	6, 65 and 70
COD, BOD, TSS, TDS	General organic/inorganic load	COD: 13 600–24 333; BOD: 1445–2803; TSS: 1033–3216; TDS: 26 166–49 996	Oxygen depletion; water quality deterioration	Algal consortia can reduce COD/BOD >60% under optimal conditions	65 and 71
Nickel (Ni)	Process chemicals	0.01–2.8	Carcinogenic; affects metabolism	Ni removal tested with *Chlorella pyrenoidosa* and *Scenedesmus* sp.	32, 65 and 72

### 2.8 The need for integrating microalgae with other technologies for TWW treatment

TWW is an issue of concern due to its complex composition and high levels of pollutants. High organic content, such as proteins, fats, and other organic matter, causes high BOD and COD. This removes oxygen from receiving water bodies, which is harmful to aquatic organisms. The presence of salt and other dissolved substances raises the levels of TDS, increasing salinity, making the water unsuitable for human and agricultural use. These elevated levels disrupt conventional treatment processes. Phenolic compounds, together with toxic elements such as Cr, Pb, Ni and other heavy metals, pose serious environmental risks. These contaminants are persistent, bioaccumulative, and harmful to the biodiversity of water bodies.

Proper treatment of tannery effluents requires an interdisciplinary solution in an efficient way, linking advanced technologies such as bioremediation, oxidation processes, and constructed wetlands. The recovery of resources and the reduction of harmful chemicals are fundamental to ensure industrial sustainability and, consequently, environmental and human health. While microalgae offer significant potential in treating TWW due to their ability to uptake nutrients, heavy metals, and organic pollutants, their standalone application faces several limitations when dealing with complex effluents like TWW.^73,74^ TWW typically contains high concentrations of toxic substances such as trivalent and hexavalent chromium, sulfides, surfactants, and persistent organic pollutants, which can inhibit microalgal growth and reduce treatment efficiency. Moreover, extreme pH values, high salinity, and low biodegradability further challenge the performance of microalgae in isolation.

**Fig. 2:**
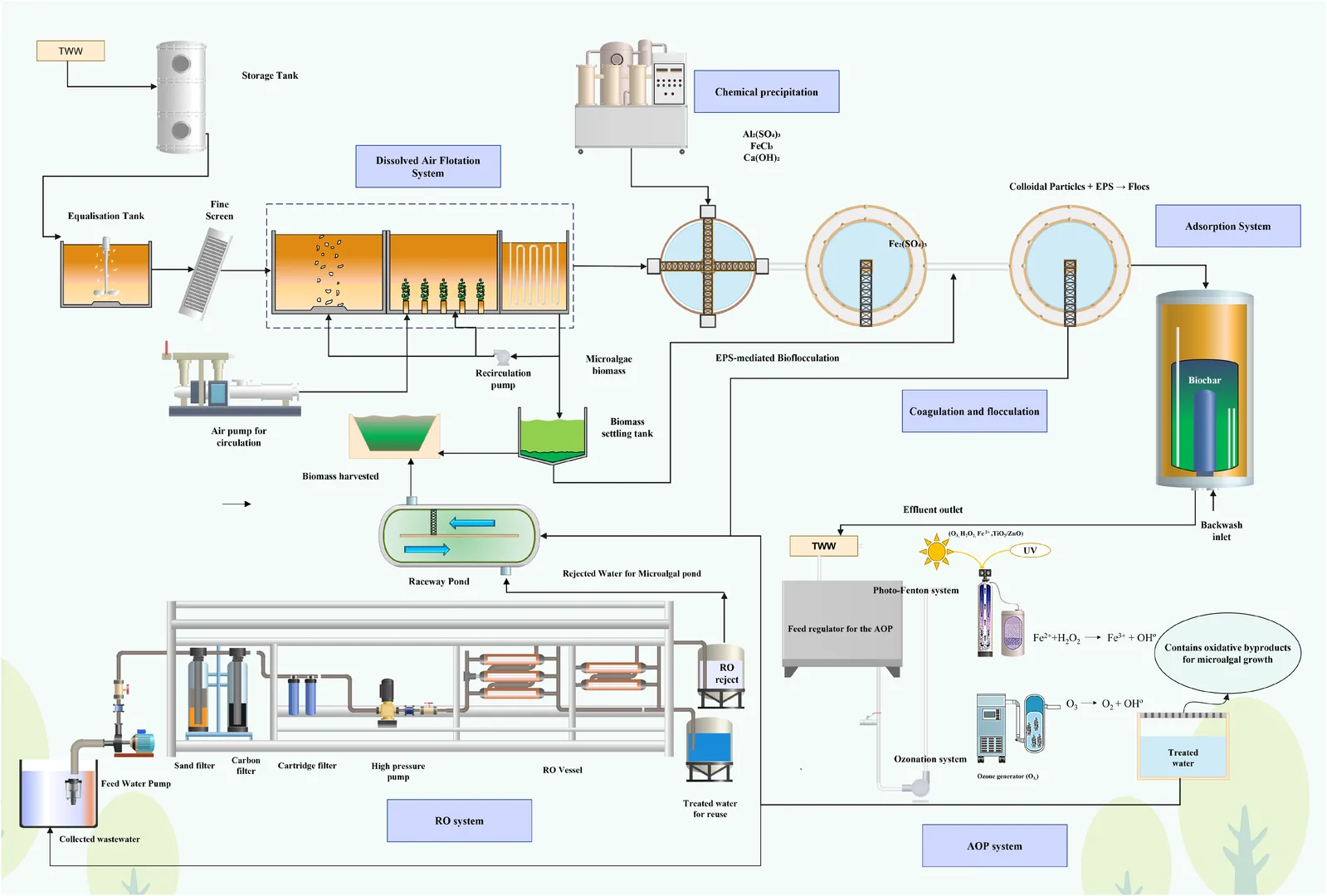
Possible integration of microalgal approach into TWW system.

To overcome these challenges, microalgal treatment systems are increasingly being integrated with other physical, chemical, and biological methods. Fig. 2 provides the schematic representation of possible integration of microalgal approach into physical, chemical and biological treatment system. For instance, combining microalgae with anaerobic digestion or activated sludge processes can improve organic load reduction and nutrient recovery. Advanced oxidation processes (AOPs) and membrane filtration can be used prior to microalgal cultivation to reduce the toxicity and increase the bioavailability of pollutants such hybrid systems not only improve pollutant removal efficiency but also enable resource recovery, such as biomass for biofuel production, making the overall treatment process more sustainable and economically viable.^10^ Therefore, integrating microalgae with complementary technologies is essential for the effective and scalable treatment of TWW, particularly in real-world, high-load industrial scenarios. The varied performance of these hybrid configurations requires a organized comparison to recognize the most effective remediation routes. Table 2 consolidates the pollutant removal efficiencies reported across the different integrated treatment approaches considered in this review. This integrated data permits for a direct evaluation of how different microalgae-based systems behave against key parameters such as COD, chromium, and nutrients under diverse integration scenarios.

**Table 2:** Pollutant removal efficiencies by microalgae-based integrated systems in tannery wastewater

Species/consortium	Analyzed parameters	Removal (%)	References
*Nannochloropsis oculata* + ozonation	COD/Cr/NH_4_–N/PO_4_–P	84–100	10
*Chlorella* sp. + *Phormidium* sp. (marine)	BOD/COD/TN/Cr/TDS	58–94	75
*Chlorella vulgaris* + *Pseudochlorella pringsheimii*	Cr/COD	> 63	76
*Scenedesmus* sp.	N–NH_3_/P–PO_4_/COD	80–96	77 and 78
Native microalgal consortium (Pena *et al.*)	N–NH_3_/TKN/P–PO_4_^3−^/COD	51–99	77
*Chlorella* sp. (Yercaud isolate)	COD/BOD/Cr/Cu/Pb/Zn	64–95	21
*Chlorella protothecoides* + biochar	Cr(iii)	99.6 (synthetic)	79
*Fusarium subglutinans* (MFC)	Cr(vi)	99.9	80

## 3. Microalgae integration with physical treatment system

The tanning process generates wastewater with high levels of organic matter, suspended solids, as well as heavy metals and toxic substances, making this task complex. A hybrid approach that combines physical methods and microalgae systems can be efficient, sustainable, and effective in removing pollutants.

### 3.1 Sedimentation and dissolved air flotation

Sedimentation and flotation are standard primary treatment methods for removing suspended solids and floating oils in tannery effluents. In sedimentation, heavier particulates settle by gravity, whereas dissolved air flotation (DAF) uses microbubbles to lift lighter materials such as fats to the surface.^81^ After primary sedimentation or flotation, a significant fraction of nutrients (like nitrogen, phosphorus, and trace metals) remains in the wastewater. Microalgae possess functional groups (*e.g.*, carboxyl, hydroxyl, amino) on their cell surfaces that bind with heavy metals (*e.g.*, Cr, Zn).^13^ Some species possess metal transporter proteins or sequester metals into vacuoles or bind them to intracellular ligands like phytochelatin, reducing toxicity. Integrating microalgae post-sedimentation improves treatment performance. For instance, *Scenedesmus* sp. and *Chlorella vulgaris* have demonstrated the ability to grow in tannery effluent following sedimentation, utilizing residual nutrients and facilitating the uptake of metals like chromium and zinc.^50^

Recent advancements in integrated wastewater treatment approaches have highlighted the synergistic potential of combining microalgal cultivation with dissolved air flotation (DAF) systems, particularly in industrial effluent settings. For instance, a study demonstrated the effective use of *Chlorella sorokiniana* cultivated in wastewater, where biomass harvesting was achieved through a two-step process involving chemical coagulation followed by DAF. This approach not only facilitated high-efficiency biomass recovery but also significantly improved effluent clarity by reducing turbidity 96% and suspended solids 92%.^82^ Although the study did not specifically target TWW, the methodology is highly transferable due to the comparable complexity of tannery effluents, which are typically rich in suspended solids, heavy metals, and organic pollutants. Integrating microalgal treatment post-sedimentation, followed by DAF, presents a promising tertiary treatment strategy enhancing both pollutant removal and biomass valorization, while reducing the environmental footprint of conventional TWW treatment plants.

### 3.2 Filtration and reverse osmosis

Filtration technologies such as sand filtration and membrane processes ultrafiltration (UF) or nanofiltration (NF) are highly effective for removing residual suspended solids and particulate matter. Reverse osmosis (RO) offers molecular-scale rejection of dissolved ions and heavy metals. However, the high operating costs, energy demand, and susceptibility to membrane fouling (from colloids, organic substances, biofilms, and mineral scaling) can significantly reduce system lifespan and increase maintenance requirements.^83^ Integrating microalgae treatment before filtration and RO offers a biologically driven solution to mitigate these challenges. Multiple studies have shown that the growth of *Chlorella* spp. or other algae in pretreated effluent can substantially reduce concentrations of dissolved solids, heavy metals, COD, and BOD, improve feed water quality and ease downstream pressure on membranes.^75,78^

Mechanisms involved includes (1) biosorption and bioaccumulation: negatively charged extracellular polymeric substances (EPS) produced by algae bind heavy metal ions (Cr, Cu, Zn, *etc.*), removing them from the aqueous phase (2) organic assimilation: algae consume organic carbon, nitrogen, and phosphorus during metabolic growth, decreasing COD, BOD, and nutrient loads, which are critical contributors to membrane fouling. (3) Aggregation and floc formation: the formation of algal aggregates or EPS-associated flocs helps reduce colloidal content and fine particles that otherwise clog membranes.^13^ These pretreatment effects reduce concentration polarization and reversible/irreversible fouling common in UF and RO with lower buildup of organic and colloidal layers (foulants) on membrane surfaces.^84^

A related study combining ozonation with algal treatment showed significantly improved recovery of microfiltration membranes treating RO concentrate, where *in situ* alkaline pH (∼12) generated by photosynthetic microalgae helped clean bio fouled surfaces and restore permeability.^84^ In TWW treatment, preliminary UF/NF and RO trials have shown that RO membranes generally exhibit lower fouling rates and higher salt removal efficiency when acting on biologically pretreated effluents, but still face flux decline due to residual COD and organic substances.^85^ Microalgal pretreatment thus promises to extend membrane operational lifespan, reduce cleaning frequency, and lower cost. Furthermore, combining algal pretreatment with biofiltration in other reuse systems has been shown to extend RO membrane run-times from about 72 h to over 300 h by substantially lowering assimilable organic carbon (AOC) and dissolved organic carbon (DOC) in the feed stream.^86^ The suggested system is based on an integrated (dual) methodology, in which microalgae treatment operates as a biological pre-treatment step. It does not replace filtration and reverse osmosis (RO) but works in synergy with them. The aim of this biological phase is to mitigate organic and inorganic fouling charge before the feedwater reaches the membranes, which extends the operational life of the system and decreases the occurrence of chemical cleanings. The quantitative benefits of this configuration over the exclusive use of filtration and RO are endorsed by the literature. Studies show that the cultivation of microalgae associated with the coagulation separation process-DAF accomplishes decreases of up to 96.2% in turbidity and 92.5% in the removal of suspended solids in complex effluents.^82^ This performance declines the potential for particulate clogging in subsequent membrane units. Particularly in tannery effluents, the cultivation of *Scenedesmus* sp. in lime wastewater, the removal of ammonium nitrogen (85.6%), phosphorus (96.8%) and COD (80.3%) was reached, under optimized conditions. At the same time, algal consortium of *Chlorella* and *Phormidium* sp. reduced BOD and COD by more than 90%, in addition to removing 94.4% of chromium in raw effluent.^75^ The microalgal pretreatment step lowers the concentration of key fouling agents, such as dissolved organic matter, nutrients, and suspended solids, that would else clog the UF and RO membranes. Then, the increase in transmembrane pressure is diminished and the frequency of chemical cleaning is reduced.

The additional capital and operational costs of a biological pretreatment unit are real and considerable. Though, this economic balance tends to balance when we account for the greater strength of the membranes that reduces replacement costs, the lower demand for chemical cleaning efforts and the potential for valuing algal biomass for the co-production of biofuels, biofertilizers or pigments.

### 3.3 Potential and challenges of integration in physical treatment system

The integration of microalgae with physical treatment technologies such as sedimentation, filtration, flotation, and membrane separation offers a multi-functional approach that simultaneously enhances pollutant removal, supports biomass regeneration, and enables production of value-added coproducts (*e.g.* biofuels, biopolymers, pigments). By coupling physical separation with biological uptake, these hybrid systems can significantly reduce concentrations of organic matter, nutrients, and heavy metals.^1^ However, realizing this integration in TWW applications presents significant challenges. The harsh chemical composition of raw tannery effluent characterized by high loads of chromium, organic solvents, sulfides, fats and oils can inhibit microalgal growth, especially when heavy-metal concentrations exceed microbial tolerance thresholds.^79^ Elevated metal concentrations (*e.g.*, Cu, Cr, Pb, Zn) not only reduce growth rates but can cause complete inhibition beyond 2–12 mg L^−1^ ranges depending on species (*e.g. Desmodesmus*, *Chlorella*).^76^

The ability of microalgae to grow efficiently in extreme conditions, such as industrial wastewater, offers new, environmentally friendly, and inexpensive water treatment alternatives. Microalgae are important in the polishing phase of physical systems. Species such as *Scenedesmus* sp. and *Pseudochlorella pringsheimii* effectively remove the remaining nutrients, *e.g.* nitrogen and phosphorus, heavy metals and recalcitrant organic matter. Microalgae can be used to remove more than 80% of hexavalent chromium and more than 60% of carbon dioxide in pretreated effluents.^87,88^

## 4. Microalgae integration with chemical treatment system

Chemical treatment in tannery effluents is applied for heavy metal removal, pH balancing, precipitation of suspended solids and reduction of complex organic loads. The combination of these processes with the use of microalgae is promising, combining the efficiency of chemical treatment with nutrient absorption, heavy metal adsorption, and potential for absorption of recalcitrant organic compounds from microalgae.^75^

### 4.1 Chemical precipitation

Chemical precipitation is a commonly utilized technique in industrial wastewater treatment for the elimination of suspended solids and dissolved heavy metals. During this process, chemical reagents including aluminium sulfate (Al_2_(SO_4_)_3_), ferric chloride (FeCl_3_), and lime (Ca(OH)_2_) are introduced to the effluent to interact with metal ions, resulting in the formation of insoluble metal hydroxides or sulfates. The insoluble agglomerates subsequently settle due to gravity or are separated through flotation, facilitating the removal of contaminants such as chromium, zinc, copper, and lead.^89,90^ Lime is notably effective in precipitating hexavalent chromium (Cr^6+^) as chromium hydroxide at pH levels exceeding 8.5, whereas alum and ferric salts are commonly employed for the reduction of phosphate and turbidity.

Microalgae have been investigated as a complementary or post-treatment strategy to mitigate these drawbacks. Numerous studies indicate that microalgae, including *Chlorella* and *Scenedesmus*, can effectively improve the removal of residual heavy metals, especially chromium, after chemical precipitation. *Chlorella* sp. has been shown to eliminate up to 99% of residual chromium *via* biosorption and bioaccumulation mechanisms. *Scenedesmus obliquus* exhibited significant tolerance to post-precipitation effluents and efficiently assimilated residual Cr, Cu, and Zn ions.^75,91^ The combination of microalgae with chemical precipitation accomplishes two functions: it enhances effluent quality by eliminating residual metal ions and organic matter, while also producing algal biomass that can be converted into bioenergy, fertilizers, or other bio-based products. Additionally, the growth of microalgae in post-precipitated effluents is frequently enhanced by increased nutrient concentrations, especially phosphorus and nitrogen, which improves both remediation effectiveness and biomass yield.^92,93^

The effectiveness of hybrid systems is contingent upon the regulation of pH, residual coagulant levels, and the timing of microalgal inoculation to prevent growth inhibition. Metal toxicity, particularly from residual Fe^3+^ or Al^3+^, can adversely affect algal physiology if not adequately controlled. With suitable process optimization, the integration of chemical precipitation and microalgae presents a viable approach for sustainable treatment of TWW, reducing environmental impacts.

### 4.2 Advanced oxidation processes

Advanced oxidation processes (AOPs) represent a class of emerging treatment technologies that rely on the generation of highly reactive hydroxyl radicals (˙OH) to oxidize and decompose refractory organic compounds present in industrial effluents, including those from tanneries.^10^ These radicals possess a high oxidation potential (2.80 V), enabling the breakdown of complex molecules such as phenols, dyes, and tannins that are resistant to conventional biological degradation.^94^ Several methods exist for hydroxyl radical generation, including ozonation, UV/hydrogen peroxide, and Fenton or photo-Fenton reactions. For instance, ozone reacts with water to produce hydroxyl radicals:O_3_ + H_2_O → 2˙OH + O_2_

Similarly, ultraviolet irradiation of hydrogen peroxide leads to radical formation through photolysis:H_2_O_2_ + *hν* → 2˙OH

Another common AOP in tannery effluent treatment is the Fenton reaction, where ferrous ions catalyze the decomposition of hydrogen peroxide:^95^Fe^2+^ + H_2_O_2_ → Fe^3+^ + ˙OH + OH^−^

These processes are highly effective in reducing COD, color, and toxicity. However, they also present several challenges, including high energy consumption, generation of oxidative byproducts (*e.g.*, short-chain aldehydes and organic acids), and the need for precise pH and reagent control.^10^ Additionally, residual oxidants and intermediates can persist in the treated water, requiring further polishing to meet discharge standards.

To address these limitations, the incorporation of microalgae as a post-AOP polishing stage offers a sustainable and synergistic solution. Microalgae such as *Scenedesmus* sp. and *Chlorella* sp. can assimilate the residual nutrients (*e.g.*, nitrates and phosphates) and intermediate organic compounds remaining after AOP treatment. The nutrient assimilation by microalgae occurs *via* biologically mediated transformations.^50^ For example, nitrate reduction in algal cells follows a sequence that includes enzymatic conversion to ammonium, ultimately incorporated into amino acidsNO_3_^−^ → NO_2_^−^ → NH_4_^+^ → biomass

Likewise, phosphate is taken up through active transport mechanisms and incorporated into structural and metabolic biomolecules such as ATP and nucleic acids:PO_4_^3−^ → cellular phosphates

Furthermore, low-molecular-weight organic acids (*e.g.*, acetic acid) generated from AOPs can serve as carbon sources for mixotrophic or heterotrophic growth in microalgae, enhancing biomass productivity while further reducing COD. During photosynthesis, microalgae also generate oxygen:6CO_2_ + 6H_2_O + Light → C_6_H_12_O_6_ + 6O_2_

This biologically produced oxygen can further support oxidative degradation of residual pollutants or enhance aerobic microbial activity in subsequent treatment stages. Ajayan *et al.* (2015) demonstrated that *Scenedesmus* sp. significantly reduced residual COD and nutrient concentrations in post-AOP tannery effluent, showcasing the potential of algal integration for enhanced polishing.^50^ However, care must be taken to neutralize or remove residual oxidants (*e.g.*, H_2_O_2_, O_3_) that could inhibit algal growth, and to optimize environmental conditions (pH, light, CO_2_) for effective microalgal performance.

In summary, the coupling of AOPs with microalgal post-treatment creates a complementary treatment that leverages the oxidative degradation capacity of AOPs and the assimilative, regenerative capabilities of algae. This hybrid approach not only ensures higher pollutant removal efficiency but also supports circular economy strategies through biomass valorization, offering a promising route for sustainable TWW remediation.

### 4.3 Coagulation and flocculation

The application of ferric sulfate (Fe_2_(SO_4_)_3_), cationic polymers, and various chemical agents contributes significantly to the coagulation–flocculation process. The application of these reagents leads to the destabilization of surface charges on colloidal particles, facilitating their aggregation into flocs that can subsequently be separated. A typical reaction of ferric sulfate with water is as follows:Fe_2_(SO_4_)_3_ + 6H_2_O → 2Fe(OH)_3_ ↓ + 3H_2_SO_4_In this context, Fe(OH)_3_ functions as a sweep floc, effectively adsorbing and entrapping suspended solids and organic materials. Nonetheless, the excessive application of these coagulants elevates the salt concentration in the effluent, potentially polluting water quality.^96–98^ Microalgae have been recognized as natural bioflocculants, presenting a sustainable alternative. Specific species such as *Chlorella* sp. and *Phormidium* sp. produce extracellular polymeric substances (EPS), functioning as biopolymers that facilitate the binding of fine particles. The streamlined process of EPS-mediated bioflocculation is as follows:Colloidal Particles + EPS → FlocsIn some studies, the co-cultivation of *Phormidium* sp. and *Chlorella* sp. resulted in more than 90% BOD and COD removal, significantly reducing solids and suspended nutrients. Additionally, the biomass produced during this process can be easily harvested and repurposed, enhancing sustainability.^75,99^ Pena *et al.* (2019) investigated the application of a microalgal consortium for the treatment of tannery effluents, yielding promising outcomes. The consortium, derived from a decommissioned effluent treatment decanter, underwent testing across a range of wastewater streams, spanning from wet to finished leather processing.^77^ Following a duration of 16 days, the biomass reached a maximum concentration of 1.77 g L^−1^ after the primary coagulation and flocculation processes. The observed removal efficiencies were as follows: 99% for N–NH_3_, 82% for TKN, 91% for P–PO_4_^3−^, and 51% for COD. While growth was not observed in 100% raw effluent, the combination of 50% raw effluent with 50% biologically treated effluent facilitated substantial biomass yields (1.3 g L^−1^) and effective nutrient removal.^77^

### 4.4 Adsorption

The process of adsorption demonstrates significant efficacy in the elimination of dyes, heavy metals, and organic compounds present in TWW. The process entails the attachment of impurities to the surface of adsorbent materials. The overarching mechanism can be depicted asM^*n*+^_(aq) + Adsorbent → Adsorbent − M^*n*+^

M^*n*+^ denotes metal ions including Cr^3+^, Pb^2+^, or Zn^2+^. Conventional adsorbents such as activated carbon demonstrate efficacy; however, they present challenges in terms of cost and regeneration difficulties. Microalgae, particularly *Chlorella vulgaris*, present a bio-based approach, characterized by cell wall functional groups (*e.g.*, –COOH, –OH, –NH_2_) that facilitate metal binding.^12^Algal–COO^−^ + Cr^3+^ → Algal–COOCr^2+^In the case of Cr(vi) removal, a two-step mechanism is observed:

(1) Reduction of Cr(vi) to Cr(iii):Cr_2_O_7_^2−^ + 14H^+^ + 6e^−^ → 2Cr^3+^ + 7H_2_O

(2) Precipitation of Cr(iii):Cr^3+^ + 3OH^−^ → Cr(OH)_3_↓

The immobilization of *Spirulina* sp. with silica gel has demonstrated significant efficacy in the removal of Cr(vi), with nearly complete adsorption achieved within a 60-minute timeframe at an optimal pH of 3.^100^ In a similar trial, Venkatesan and Sathiavelu (2022) investigated *Chlamydomonas moewusii* (SMA1), *Scenedesmus* sp. (SMA2), and *Auxenochlorella pyrenoidosa* (SMA3) with respect to their efficacy in chromium removal from tannery effluent. SMA2 demonstrated the most significant biosorption efficiency at 90%, with SMA3 following at 80% and SMA1 at 65%.^101^

The kinetics and capacity of microalgal adsorption are often modeled by the pseudo-second-order kinetic model:
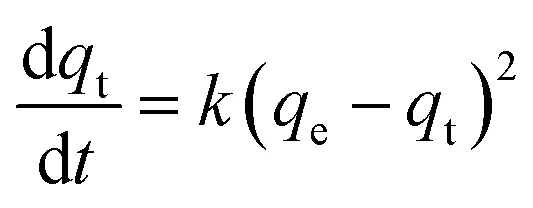
And the Freundlich isotherm:
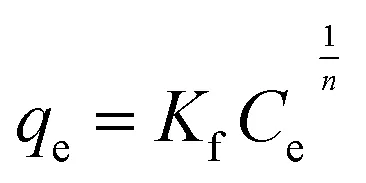
where *q*_t_ is the adsorption capacity at time *t*, *q*_e_ is equilibrium capacity, *C*_e_ is equilibrium concentration, *k* is the rate constant, and *K*_f_, *n* is Freundlich constants.

Biochar has been utilized as a preliminary treatment to improve the growth of microalgae and facilitate the removal of metals. For example, Sforza *et al.* (2020) illustrated that commercial biochar achieved a removal rate of 99% for Cr(iii) within 5 minutes, whereas pinewood biochar only reached 83% in a duration of 3 hours. The methodology adhered to the Freundlich isotherm model and conformed to pseudo-second-order kinetics. Nonetheless, the efficacy of biochar is influenced by the intricate nature of real TWW.^79^

### 4.5 Potential and limitations

The integration of microalgae into chemical treatment processes presents complementary advantages: it improves the efficiency of pollutant removal, decreases the need for chemicals, and produces valuable biomass. However, obstacles persist owing to the hazardous characteristics of tannery effluents, especially their elevated levels of heavy metals and variable pH values, which may impede the growth of microalgae. In response to these issues, it is advisable to implement strategies including pre-dilution, pH regulation, and the utilization of microalgal consortia. Additionally, the application of chemical pretreatments involving adsorbents such as biochar has been shown to mitigate toxicity and improve the resilience of algae. The integration of microalgae with chemical methods such as coagulation, precipitation, and adsorption presents a more eco-efficient and cost-effective approach to wastewater treatment.^54,102,103^ These hybrid systems present significant potential as sustainable alternatives to traditional processes. Their implementation leads to a decrease in sludge production, facilitates the recovery of resources, and aligns with the objectives of a circular bioeconomy. Nonetheless, the expansion of these systems necessitates additional investigation into optimization, variability of effluents, and the stability of long-term operations.

## 5. Integrated bio-chemical approaches for advanced tannery wastewater treatment

From an applied perspective, integrated treatment processes combining physical, chemical, and biological steps have shown rapid reduction in pollutant concentration, especially heavy metals present in tannery effluent. From several research studies, hybrid systems that combine microalgae with chemical pretreatment or hybrid chemical or algal systems have demonstrated significant synergistic removal effects.^104^ For example, advanced oxidation processes (AOPs), which include ozone, UV/H_2_O_2_, and photo-Fenton, generate hydroxyl radicals to oxidize persistent organics and partially detoxify heavy metals. This pretreatment lowers COD and improves biodegradability. Many review studies highlight that coupling AOPs with microalgae enhances overall treatment efficiency and enables biomass valorization.^73,105,106^

For example, *Dunaliella salina* achieved around 66% Cr(vi) uptake under optimal conditions of AOP pretreatment.^73,107^ In addition, coupling ozonation with phycoremediation using *Nannochloropsis oculata* led to notable improvements in water quality by reducing color, odor, COD, NH_4_^+^–N, PO_4_^3−^, Cr, and inorganic carbon levels. Ozonation as a pretreatment can break down recalcitrant organics and reduce toxic load, creating favorable conditions for subsequent phycoremediation. In a lab-scale study, TWW treated first with ozone has achieved 60% COD removal, followed by treating with *Nannochloropsis oculata*, which achieved up to 97% Cr removal, along with ammonia, phosphate, color, and odor reduction (Saranya and Shanthakumar,^10^ 2020; Satpati *et al.*,^108^ 2023). Ozonation in this context served as a pre-treatment step, partially oxidizing organic matter and facilitating algal uptake for further bioremediation (Saranya and Shanthakumar, 2020). This supports a hybrid approach in TWW treatment that pairs advanced oxidation with biological remediation.

In a study, biochar-amended plant-microbial consortia accelerated the co-removal of heavy metals such as Cr, Ni, Pb, and polycyclic aromatic hydrocarbons (PAHs) (Sarma *et al.*,^109^ 2019). The combination of *Herbaspirillum* spp. with organic acids like gluconic acid has also significantly increased metal uptake and stress tolerance in crop systems, which may be applicable in phyco-treatment for TWW treatment (Govarthanan *et al.*,^110^ 2016). Diluting concentrated tannery effluent with harvested rainwater reduced toxicity and improved subsequent microalgal phycoremediation. In a sequential phycoremediation setup using algae such as *Oedogonium* and *Pithophora*, dilution-enhanced heavy metal uptake by both microalgae and attendant macrophytes. This demonstrates the value of combining simple chemical treatment with biological treatment to achieve successful bioremediation (Mahtab *et al.*,^111^ 2024). Granulating microalgae onto carbon-activated supports, such as *Chlorella vulgaris* and *Scenedesmus obliquus* granules, provides physical adsorption sites and biomass carriers. In a study, using 9 g L^−1^ of these granules for 120 hours achieved up to 9.3 mg per L Cr removal and decreases in COD of 38%, BOD, and TSS from TWW (Mirza *et al.*,^112^ 2021). Moreover, additives like calcium ions and dipicolinic acid have been shown to enhance the metabolic activity of hydrocarbonoclastic, metal-resistant bacteria under multi-stress conditions, including heat and metal toxicity (Radwan *et al.*,^113^ 2017). Similarly, integrating chitin with sulfate-reducing bacteria (SRB) has resulted in the successful removal of sulfates and heavy metals from mine-influenced waters, suggesting parallel applicability in tannery effluents treatment (Rodrigues *et al.*,^114^ 2019). Furthermore, glucose, EDTA, and surfactants like Tween 80 have been employed to boost bioremediation efficacy to stimulate microbial metabolism or partially degrade pollutants, thereby facilitating microbial action by chemical additives (Dong *et al.*,^115^ 2013; Park *et al.*,^116^ 2011).

Although several integrated treatment strategies have demonstrated good remediation performance, their suitability depends strongly on tannery wastewater composition. The selection of an integrated treatment strategy for tannery wastewater should be based on the dominant pollutant profile and treatment objectives. For tannery effluents characterised by high concentrations of recalcitrant organic compounds, colour, and elevated COD, AOP–microalgae systems offer significant advantages because oxidative pretreatment reduces toxicity and improves the bioavailability of residual organics for subsequent phycoremediation.^73,107^ In contrast, tannery wastewaters dominated by chromium and other heavy metals are more effectively treated using adsorption-assisted microalgal systems or microalgae–fungi consortia, where extensive biosorption, ion exchange, extracellular polymeric substance (EPS)-mediated immobilisation, and intracellular sequestration collectively enhance metal removal. Among the biological approaches, microalgae–fungi consortia appear particularly promising for tannery applications because fungal pelletisation simultaneously facilitates chromium adsorption, degradation of tannins and dyes through extracellular enzymes, and simplified biomass harvesting.^117,118^ Microalgae–bacteria and microalgae–microalgae consortia are better suited for secondary treatment stages where residual nitrogen, phosphorus, and biodegradable organic matter remain after primary detoxification. Meanwhile, dilution-assisted phycoremediation can serve as a preliminary conditioning step for highly concentrated tannery effluents where excessive salinity and metal toxicity inhibit direct biological treatment.^50,111^ Considering the complex and variable composition of tannery wastewater, no single technology is likely to achieve complete remediation under all conditions. Instead, a sequential treatment by integrating physicochemical detoxification with consortium-based phycoremediation is expected to provide the most robust and sustainable solution. Given the variability in experimental setups across the literature, it is fundamental to create the environmental conditions that promote greatest remediation efficiency. Table 3 summarizes the key improved operational parameters defined in different studies, serving as a consolidated reference to support in the project and benchmarking of future combined microalgal treatment systems specifically targeting tannery effluents.

**Table 3:** Optimized operational parameters reported for microalgae cultivation in tannery and industrial wastewater

Species/consortium	Effluent/operational condition	Effluent conc. (%)	Light (µmol photons per m^2^ per s^1^)	pH	Biomass/Productivity	Lipids (%)	References
*Scenedesmus* sp.	TWW (beamhouse)	88.4	182.5	—	0.90 g L^−1^	—	78
*C. vulgaris* + *P. pringsheimii*	TWW (sewage diluted)	30	35	7.0–9.3	3.5 g L^−1^	25.4	87
*Nannochloropsis oculata*	Ozonated TWW (90 min)	100	75	7.5–8.5	0.86 g L^−1^	—	10
*Chlorella* sp.	TWW (photo-bioreactor)	90	—	Neutral	0.95 g L^−1^	—	21
*Chlorella protothecoides*	Biochar pre-treated TWW	100	—	—	Increased growth	—	79
*Spirulina* sp.	TWW (Cr adsorption)	—	—	3.0	—	—	100
*Phormidium* sp. + *Chlorella* sp.	TWW (10 mg L^−1^ initial Cr)	100	—	—	Biofilm growth	—	78

## 6. Synergistic approach for tannery wastewater treatment with microalgal microbial consortia

TWW is a complex effluent containing high concentrations of heavy metals, along with various organic and inorganic pollutants. Addressing such multifaceted contamination requires integrated strategies that use the combined synergistic approach of various microorganisms and chemicals. Among these, synergistic interactions between heavy metal-resistant microorganisms and other functional components have shown efficient results in enhancing pollutant removal more than the capability of individual organisms.^119^ Microbial consortia comprising diverse species, including bacteria, fungi, and microalgae, have demonstrated superior resilience and detoxification capabilities under metal stress compared to treatment with single species.^117^ These consortia often exhibit protocooperative behaviour, where mutualistic interactions improve tolerance to a wide range of heavy metals and pollutants to achieve higher removal efficiency. Studies indicate that co-cultivating microalgae with bacteria significantly improves the removal of heavy metals and nutrients from industrial effluents, including TWW.^120^ For example, a consortium of *Bacillus pakistanensis* with *Cladophora glomerata* has shown more than 90% of removal for metals like Cr, Pb, and Ni from industrial wastewater.^121^ This suggests similar consortia could enhance Cr, Pb, and Ni detoxification in TWW through cooperation of metabolic pathways and nutrient recycling.^122^ Co-cultivation of microalgae and fungi proves effective in adsorbing and sequestering heavy metals from aqueous systems. The fungal–algal consortia are mediated by passive adsorption onto EPS and active intracellular uptake, thereby enhancing overall metal sequestration for complete detoxification.^123,124^ Specific consortia such as *Chlorella vulgaris* with *Aspergillus* spp. have been shown to flocculate simultaneously for nutrient and heavy metal removal, which proves the consortium approach is an effective strategy for TWW.^125^ Additionally, microalgal–fungal aggregates have been employed for simultaneous biomass production and wastewater treatment, such as the bioflocculation of *Chlorella vulgaris* with *Aspergillus* spp., producing pellets that facilitate both nutrient removal and bioenergy recovery.^126^ Further, co-cultivation of fungi like *Penicillium aculeatum* and *Trichoderma* spp. has been found to enhance bioaccumulation of heavy metals such as arsenic, lead, and zinc, while simultaneously promoting growth for potential bioenergy applications.^127^ The fungus *Fusarium subglutinans*, combined with cactus-derived biosorbents, removed up to 98–99% of Cr(vi) from real tannery effluents under optimized conditions of pH 2- and 60-min contact time.^128^ This shows that fungal biosorbents, when integrated into a treatment process, can remove high concentrations of heavy metals from tannery streams. Still, there are many studies for consortium approaches with different strains and species of bacteria, fungi, and algae that are under research for the bioremediation of heavy metals and pollutants from TWW.

### 6.1 Microalgae–bacteria consortium (MBC)

**Fig. 3:**
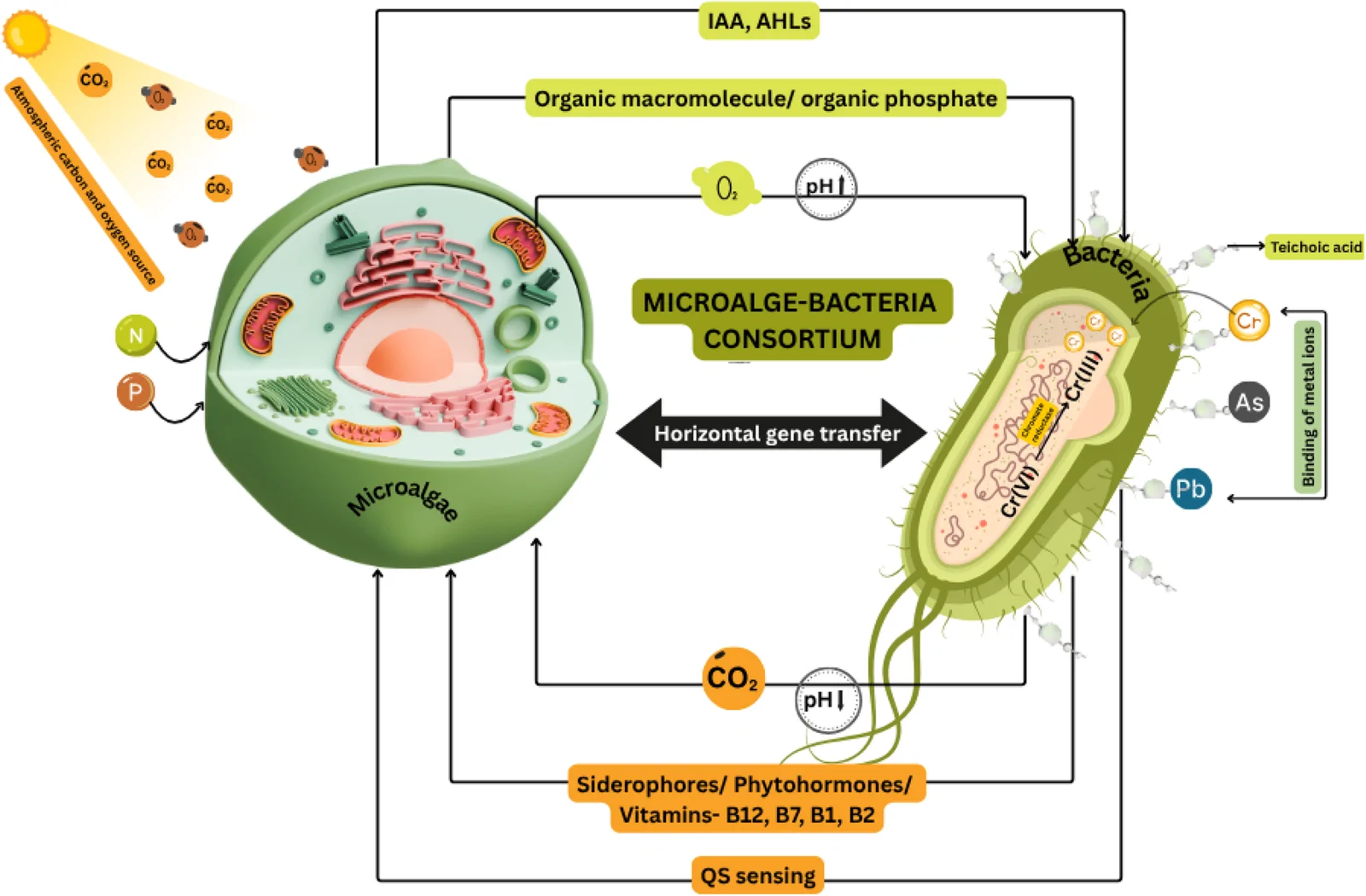
Mechanism of microalgae–bacteria consortium.

Interactions between microalgae and bacteria can take the form of symbiosis, mutual benefit, or competition as shown in Fig. 3. Mixed populations (co-culture or consortia) can perform functions that are difficult or even impossible for individual strains or species, and those that require multiple steps.^129^ A consortium may provide robustness to environmental fluctuations, stability for the strain, the ability to share metabolites, overcome nutrient limitations, and resistance to invasion by other species. There have been many research studies on the consortia of microalgae–bacteria for pollutant removal from TWW and they have shown that consortia of algae and bacteria are more efficient in wastewater treatment and nutrient recovery compared to single algal or bacterial systems.^50,87,101^ Microalgae–bacteria consortia (MBCs) offer a promising, ecologically robust, and sustainable approach to treat TWW. By integrating the photosynthetic capability of microalgae with the degradative and nutrient-cycling potential of heterotrophic bacteria, MBCs function as a biologically self-sustaining approach capable of removing a wide range of pollutants and can also produce valuable biomass and sequester carbon dioxide.

The functional interplay between microalgae and bacteria in these consortia is metabolically synergistic.^130^ Microalgae, through photosynthesis, generate oxygen, which is utilized by bacteria to oxidize organic pollutants present in TWW. In turn, bacterial respiration releases carbon dioxide, which microalgae use as a carbon source for continued growth and biomass production. This closed-loop oxygen–carbon cycle significantly reduces the need for mechanical aeration and chemical dosing, particularly important for treating effluent with high levels, such as those from tanneries.^131^ Additionally, bacteria mineralize organic nitrogen and phosphorus compounds into forms that are readily assimilated by microalgae. Consequently, this enhances the nutrient recovery and promotes further microalgal proliferation. These interactions not only improve treatment efficiency but also reduce operational costs and environmental impact.

Furthermore, microalgae and bacteria are not only limited to nutrient cycling but also engage in complex biochemical exchanges during the consortium treatment. Many microalgae are auxotrophic for essential vitamins such as B_12_, thiamine, and biotin.^132^ These micronutrients are often synthesized and released by associated bacteria, which in turn benefit from access to organic compounds produced by microalgae. Additionally, bacterial secretion of phytohormones such as indole-3-acetic acid (IAA) has been shown to stimulate microalgal growth, chlorophyll production, and photosynthetic efficiency.^133^ Such interactions are particularly beneficial in complex wastewater environments where pollutant-induced stress can inhibit microalgal growth. In addition to metabolic exchanges, microalgae and bacteria communicate through quorum-sensing (QS) molecules, including *N*-acyl homoserine lactones (AHLs), oligopeptides, and autoinducer-2 (AI-2).^134^ These signals regulate biofilm formation, enzyme secretion, and nutrient assimilation, especially within the phycosphere, a microscale zone around algal cells densely populated by bacteria.^135^ QS can promote consortium stability and functionality, and it also contributes to antagonistic interactions. Certain bacteria, such as *Kordia algicida*, secrete algaecidal proteases in a QS-dependent manner, which can inhibit or lyse algal cells.^136^ Therefore, microalgal or bacterial strain selection and its culture conditions are necessary to maintain the mutualistic cooperation to achieve stable MBC in TWW treatment systems.

One of the most promising structural configurations of MBCs is the formation of granular aggregates. These compact, spheroidal clusters which ranges from 100 to 5500 µm are stabilized by EPS composed of proteins and polysaccharides. Granular MBCs offer several advantages, including improved biomass retention, enhanced surface area for sorption, and increased resistance to shear forces.^137^ Moreover, EPS matrices protect the microbial community from heavy metal stress, such as those caused by chromium, cadmium, nickel and lead which are present in high concentration in TWW.^138^ Studies have demonstrated that co-culturing *Chlorella* with bacterial strains like *Bacillus firmus* or *Beijerinckia fluminensis* significantly improves algal tolerance to copper and enhances biomass production, pigment synthesis, and pollutant removal efficiency.^139^ Mechanistically, MBCs facilitate the removal of various pollutants through enzymatic and physicochemical processes. Organic pollutants such as dyes, surfactants, and phenolics are degraded *via* the action of lignin peroxidase (LiP), manganese peroxidase (MnP), azoreductase, and laccase enzymes secreted by bacteria and algae.^140^ These enzymes oxidize complex molecules into smaller, less toxic intermediates, which are subsequently mineralized. Meanwhile, heavy metals such as Cr(vi), Pb(ii), and Ni(ii) are removed through biosorption to functional groups on microbial cell walls and EPS, as well as through enzymatic reduction to less toxic forms. The biosorption process is facilitated by the abundant carboxyl, phosphoryl, and sulfhydryl groups on the surfaces of both bacterial and algal cells.^13^

Performance evaluations of MBCs in treating TWW reveal high removal efficiencies for COD up to 99%, nitrogen up to 98.8%, phosphorus up to 95%, and heavy metals removal of more than 90%. Biomass yields typically range from 300 to 1000 mgper L per day, with the potential for further valorization into biofuels, biofertilizers, or bioplastics.^141^ Additionally, MBCs demonstrate enhanced resilience in the presence of heavy metals, with studies showing improved tolerance and stable system operation under such stress. Importantly, these systems also contribute to greenhouse gas mitigation by capturing and utilizing CO_2_ released during organic matter degradation, thereby reducing the carbon footprint of the treatment process. The applicability of MBCs extends across various reactor configurations, including high-rate algal ponds (HRAPs), raceway photobioreactors, and aerobic granular sludge systems.^142^ Each offers specific advantages depending on the wastewater characteristics and treatment needs. HRAPs and raceways are particularly suited for large-scale applications due to their low cost and ease of operation, while AGS systems provide high-density biomass retention and superior pollutant removal in compact setups. Moreover, synthetic ecology and metabolic modeling approaches are being increasingly used to design optimized MBCs with predictive capabilities, enabling tailored solutions for specific wastewater profiles.^143^ Future research should focus on high-throughput strain selection, pilot-scale validation, and integration with biomass valorization technologies to fully harness the potential of MBCs in industrial wastewater treatment, including tanneries.

### 6.2 Microalgae–microalgae consortium (MMC)

**Fig. 4:**
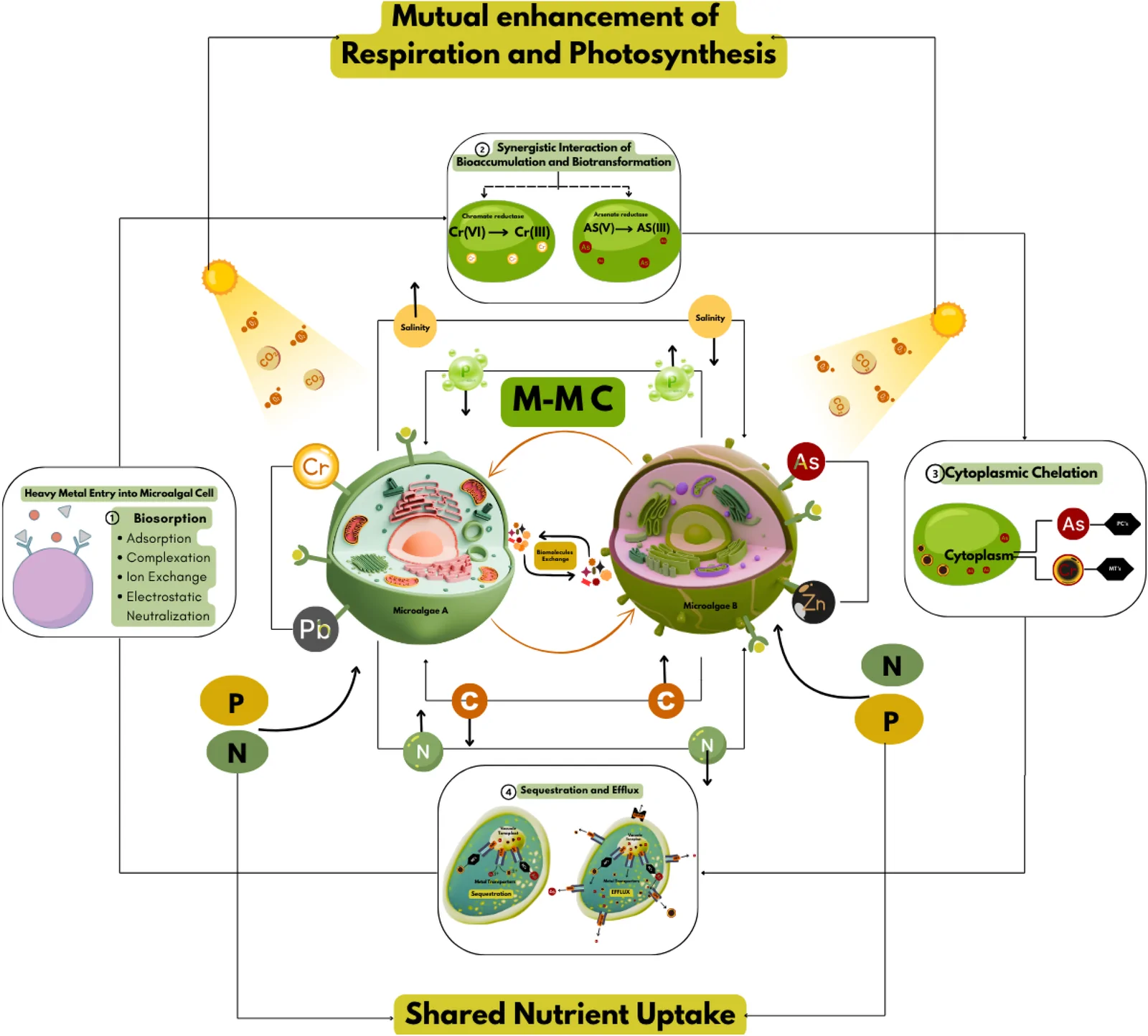
Mechanism of microalgae–microalgae consortium.

Microalgae–microalgae consortia (MMC) use the synergistic interactions between different microalgal species to enhance pollutant removal from TWW by counteracting oxidative stress caused by pollutants.^117,144^ The algal biomass produced out of the TWW treatment will pave the way for gaining valuable bio-products, thus in turn leading to a circular economy.^145^ These consortia combine the strengths of different microalgal species that are specific for heavy metal uptake, nutrient assimilation, detoxification, and antioxidant defences.^146,147^Fig. 4 illustrates the mechanistic framework that shows pollutant detoxification and nutrient exchange within microalgae–microalgae consortia. Heavy metal removal begins with biosorption, where extracellular polymeric substances and negatively charged functional groups present on the microalgal cell surface facilitate adsorption, ion exchange, complexation, and electrostatic interactions with dissolved metal ions. Chromium, one of the major contaminants in tannery wastewater, is initially contacts at the cell surface and subsequently transported into the cell, where chromate reductase enzymes catalyse the reduction of toxic Cr(vi) to the less toxic Cr(iii). Following reduction, Cr(iii) is detoxified through intracellular chelation by metal-binding biomolecules and sequestration within intracellular compartments, thereby limiting its interaction with essential metabolic pathways (Chugh *et al.*,^148^ 2022; Perales-Vela *et al.*,^149^ 2006; Tripathi *et al.*,^150^ 2024). Additionally, the fate of chromium within microalgae–microalgae consortia involves a multistep detoxification pathway that extends beyond the simple reduction of Cr(vi) to Cr(iii). Initially, dissolved chromium species are intercepted by EPS and negatively charged functional groups on the cell surface. This extracellular adsorption step reduces chromium mobility and limits its direct interaction with cellular components. Following adsorption, a fraction of Cr(vi) enters the cell through non-specific sulfate transporters due to its structural similarity to sulfate ions.^151^ Once internalised, Cr(vi) undergoes enzymatic reduction mediated by chromate reductases and intracellular reducing equivalents including NAD(P)H, glutathione, ascorbate, and other redox-active metabolites. This reduction converts highly soluble and toxic Cr(vi) into Cr(iii), thereby substantially lowering its oxidative potential and cellular toxicity. However, Cr(iii) remains potentially harmful because of its strong affinity for proteins, nucleic acids, and intracellular enzymes. Consequently, further detoxification mechanisms are required. The generated Cr(iii) is rapidly complexed with intracellular metal-binding ligands, including phytochelatins, metallothionein-like peptides, glutathione, and other sulphur-rich biomolecules. These chelation reactions reduce the concentration of free chromium ions within the cytoplasm and prevent interference with critical metabolic pathways. Subsequently, chromium–ligand complexes are compartmentalised into vacuoles, where they remain biologically inactive.^152,153^ Simultaneously, a portion of Cr(iii) becomes immobilised within the EPS matrix surrounding the consortium, where hydroxyl, carboxyl, and phosphate groups provide stable binding sites that prevent remobilisation into the aqueous phase. Under alkaline microenvironments generated during photosynthetic activity, dissolved Cr(iii) may additionally precipitate as insoluble chromium hydroxide [Cr(OH)_3_], further reducing its bioavailability. Excess intracellular chromium can also be transported out of the cell through ATP-dependent efflux systems, thereby maintaining intracellular metal homeostasis. Microalgae–microalgae consortia lies in the establishment of complementary nutrient acquisition and metabolite exchange networks that enhance consortium stability under the highly stressed conditions of tannery wastewater.^76,154^

Furthermore, unlike monocultures, different microalgal species possess distinct affinities and uptake kinetics for nitrogen, phosphorus, inorganic carbon, trace elements, and dissolved organic compounds. Consequently, resource partitioning occurs within the consortium, reducing direct competition for nutrients while maximising overall nutrient utilisation efficiency. For instance, one species may preferentially assimilate nitrate through nitrate reductase-mediated pathways, whereas another species may exhibit greater affinity towards ammonium assimilation *via* the glutamine synthetase–glutamate synthase (GS-GOGAT) cycle. Such metabolic complementarity enables simultaneous exploitation of multiple nitrogen pools present in tannery wastewater.^77,155^ In addition to nutrient partitioning, interspecific metabolite exchange contributes significantly to consortium performance. During photosynthesis, microalgae continuously release extracellular organic carbon in the form of carbohydrates, amino acids, organic acids, alcohols, and low-molecular-weight metabolites. These compounds can be assimilated by neighbouring microalgal species under nutrient-limited or stress-induced conditions, thereby establishing a cross-feeding mechanism that sustains biomass productivity. Similarly, extracellular phosphatases secreted by certain microalgal species hydrolyse organically bound phosphorus into bioavailable orthophosphate, which subsequently becomes accessible to the entire consortium. This collective nutrient mobilisation improves phosphorus recovery from tannery wastewater and supports sustained growth under nutrient-fluctuating conditions.^156,157^

(Singh and Singh, 2022) investigated the consortium of *Scenedesmus vacuolata*, *Chlorococcum humicola*, and *Tetradesmus* with *Scenedesmus* for municipal wastewater treatment that achieved nutrient assimilation rates of 79–98% and heavy metal detoxification efficiencies between 50–94%.^146^ In addition, it increases the biomass yield by 1.4 to 1.6-fold and improves photosynthetic performance, which demonstrates their capability in detoxifying pollutants in TWW. Such enhanced performance by microalgae consortium is likely mediated by complementary EPS produced on the cell surface, mutual cooperation for light-harvesting, and cooperative reactive oxygen species defence mechanisms, which collectively improve the stress resilience and detoxification capacity.

In the context of TWW, mono microalgal culture has already shown promising results. (Saranya and Shanthakumar, 2019) showed that a consortium of *Chlorella vulgaris* and *Pseudochlorella pringsheimii* achieved over 80% Cr removal and 65% COD reduction at 30% effluent dilution, with biomass yields of 3.5 g L^−1^ and lipids up to 25%. Likewise, *Scenedesmus* sp. reduced heavy metals such as Cr (81–96%), Cu (73–98%), Pb (75–98%), Zn (65–98%) and phosphate (>95%) over 12 days.^10,87^ Importantly, the consortia approach has been proven effective in other wastewater, such as Sewage, Municipal, textile, and dye. A consortium of *Chlorella vulgaris* and *Scenedesmus dimorphus* culture achieved lipid contents of 28–31% in tannery-industry effluent.^77,158^ In addition, the co-cultivation of *Chlorella* and *Scenedesmus* spp. has shown high efficiency in heavy metal detoxification and nutrient assimilation. Experimental studies have reported up to 99% removal of Cr(vi) both in synthetic and real wastewater.^159^

In a study, a consortium comprising *Phormidium* sp. and *Chlorella* sp. achieved 94.45% chromium removal from tannery effluent with an initial Cr concentration of approximately 10 mg L^−1^.^75^ The development of extracellular polymeric substance (EPS)-rich biofilms by this algal consortium further enhanced the adsorption and immobilization of heavy metals, leading to notable removal efficiencies of upto 94% under both planktonic and biofilm-associated conditions.^75^ These results show the potential of microalgae–microalgae consortia as effective, sustainable alternatives for treating complex industrial effluents like tanneries. Mechanistically, an interspecies consortium provides distinct advantages. Varied nutrient uptake pathways reduce competition while collectively broadening the spectrum of metabolized contaminants. More robust EPS layer formation improves pollutant adsorption, which includes heavy metals and biofilm resilience. Additionally, coordinated antioxidant systems such as peroxidase, SOD, and catalase prevent microalgal cell from oxidative damage caused by metal stress and ROS generated during detoxification.^160,161^

Furthermore, algal biofilm reactors and immobilised consortia show greater effluent treatment and biomass density, offering improved light utilisation and easier harvesting than suspension systems.^162,163^ Reactors such as suspended pond reactors, high rate photobioreactors (PBRs), and biofilm reactors are efficient in scaling up the effluent treatment process. Suspended raceway ponds offer low-cost, large-volume treatment but face challenges in harvesting small algal cells. Photobioreactors designed as tubular, plate, or bag designs achieve higher cell density and biomass quality, though their capital costs require offset *via* biomass valorisation (*e.g.*, biofuel, bioplastics).^163–165^

However, algal biofilm or phototrophic-biofilm reactors allow microbes to attach on inert carriers like HDPE and nylon, which form EPS-rich matrices that enhance light penetration, simplify biomass recovery, and use lower energy.^155^ Moving bed biofilm reactors (MBBRs) extend this concept physically, which facilitates continuous agitation and robust biofilm development for tannery effluents, which have high heavy metal concentration.^166^ In pilot-scale applications, treating agricultural runoff using tubular PBRs reached productivity of 2–14 g TSS per m^3^ d, with high nutrient and emerging contaminant removal.^167^ These results indicate that it is feasible to implement in tannery effluent treatment to remove nutrients and heavy metals. Application in tannery contexts appears promising, though specific studies remain low. Given the complementary metabolic profiles, MMCs are expected to achieve ≥85–95% removal across COD/BOD, nutrients, and heavy metals when applied to raw TWW. In the future, research should focus on optimising species ratios, reactor configurations, and dilution strategies to maximise removal efficiency and valorise post-algal biomass for producing value-added products.

### 6.3 Microalgae–fungi consortium (MFC)

**Fig. 5:**
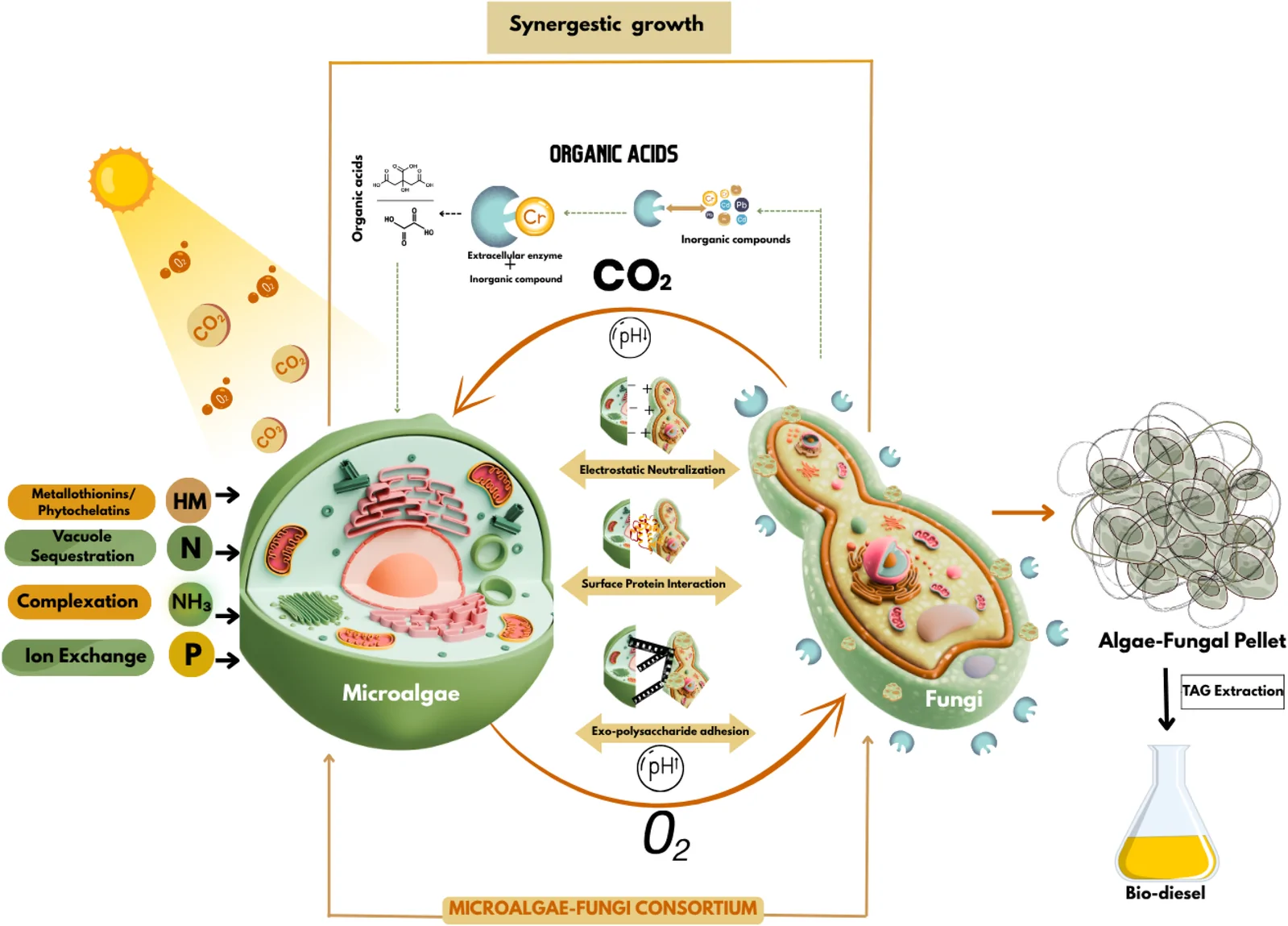
Mechanism of microalgae–fungi consortium.

Microalgae–fungi consortium harness fungal pelletization that is achieved through optimized agitation and pH control, to create bio flocculating aggregates that immobilize microalgae for enhancing surface contact to remove pollutants (Fig. 5).^168^ Electrostatic interactions, EPS matrices, and bonding *via* amino, carboxyl, hydroxyl, and phosphate groups facilitate strong physical adhesion and enable the formation of stable consortium biofilms.^169^ Mechanistically, fungi contribute extracellular enzymes to degrade complex organics in tannery effluents such as tannins and dyes.^170^ In addition, it also solubilizes suspended solids and liberates CO_2_ through heterotrophic metabolism. The CO_2_ liberated from fungi is consumed by microalgae to be re-assimilated phototrophically. Consequently, it generates oxygen that supports further fungal respiration and growth. Simultaneously, microalgae assimilate nitrogen and phosphorus, maintaining nutrient balance and pH stability.^171^

The pollutant removal capability of microalgae–fungi consortia is facilitated by a cascade of extracellular and intracellular detoxification mechanisms. Initially, fungal hyphae and microalgal cell surfaces provide abundant binding sites through polysaccharides, proteins, lipids, and extracellular polymeric substances (EPS), which contain negatively charged carboxyl, hydroxyl, phosphate, and amino functional groups. These facilitate the adsorption of dissolved metal ions through electrostatic attraction, surface complexation, and ion-exchange reactions.^169^ During ion exchange, native ions associated with fungal and algal cell wall matrices, including H^+^, Na^+^, K^+^, Ca^2+^, and Mg^2+^, are displaced by chromium, nickel, lead, zinc, and other heavy metals present in tannery wastewater. Simultaneously, complexation reactions immobilise metals through the formation of stable coordination bonds with oxygen-, nitrogen-, and sulfur-containing ligands distributed throughout the biofilm matrix.^77,172^

Following extracellular immobilisation, a fraction of dissolved chromium enters fungal and algal cells through non-specific sulfate transport systems and metal transport proteins. Intracellularly, Cr(vi) undergoes enzymatic reduction mediated by chromate reductases, glutathione-dependent reductases, NAD(P)H-dependent oxidoreductases, and other redox-active metabolites, generating the less toxic Cr(iii) species. However, Cr(iii) can still interfere with cellular proteins and nucleic acids if not further detoxified. Consequently, the consortium activates metal sequestration pathways involving phytochelatins, metallothioneins, glutathione, and cysteine-rich peptides. These sulfur-containing ligands strongly coordinate Cr(iii), forming stable chromium–ligand complexes that are subsequently compartmentalised within vacuoles and intracellular vesicles, thereby preventing interference with photosynthesis, respiration, and cellular metabolism.^173,174^

Fungal metabolism further strengthens this detoxification network through the secretion of extracellular oxidoreductases, laccases, manganese peroxidases, lignin peroxidases, and hydrolases capable of degrading tannins, azo dyes, phenolic compounds, and other recalcitrant organics present in tannery wastewater. The degradation process generates low-molecular-weight organic acids such as oxalic, citric, gluconic, and malic acids, which alter metal speciation and improve metal accessibility for subsequent biosorption and bioaccumulation.^175^ At the consortium level, fungal respiration continuously supplies CO_2_ that is assimilated by microalgae through photosynthesis, while oxygen released by microalgae sustains fungal aerobic metabolism. In parallel, fungal mineralisation of organic matter releases inorganic nitrogen, phosphorus, trace minerals, and micronutrients that are readily assimilated by microalgae. This bidirectional exchange of carbon, nutrients, and metabolic intermediates establishes a self-sustaining mutualistic network that enhances biomass productivity, pollutant degradation, heavy metal immobilisation, and overall treatment efficiency.^176^ Moreover, fungal pelletisation provides an important structural advantage within the consortium. The three-dimensional hyphal network functions as a biological scaffold that immobilises microalgal cells, increases biomass retention, improves light distribution, and enhances mass transfer between consortium partners. The resulting algal–fungal pellets possess significantly higher adsorption capacity, greater resistance to heavy metal toxicity, and improved harvesting efficiency compared with suspended monocultures.

A study used *Aspergillus fumigatus* pellets with *Synechocystis* sp. PCC6803, achieving 98% immobilisation efficiency and cadmium adsorption of 98.9% (37.3 mg g^−1^) which shows MFC's potential for strong heavy metal uptake synergy.^177^ Similarly, *Fusarium subglutinans* biomass removed 99.9% Cr(vi) at pH 2 in 60 minutes, illustrating fungi's high metal tolerance and rapid biosorption capability.^80^ Whereas *A. terreus* immobilized on sodium hydroxide–treated biomass similarly removed Cr(vi) efficiently from TWW.^178^ A consortium of fungal–algal systems has shown higher efficiency compared to monocultures in removing pollutants.^155^ For example, consortium of *Aspergillus niger* pellets and *Chlorella vulgaris* treated municipal wastewater which reduces COD by 83.7%, BOD by 81.8%, and TOC by 70.3% within 24 h and increasing biomass by 29% and lipid content by 26%.^125^ A consortium of *Penicillium* sp. and *Chlorella* sp. achieved upto 96.5% and 96.1% removal of COD and BOD, 92.5% and 94.1% removal of nitrate and ammonia, and complete removal of phosphate from livestock wastewater.^179^ In sugar mill sludge treatment, a tri-culture with *Chlorella*, *Chaetomium gracile*, and *A. niger* removed 83.5% TP, 94.5% TN, and 76.9% COD alongside producing 5.75 g L^−1^ biomass.^180^ Similarly, fungal–microalgal symbiotic systems can also be established by introducing fungal mycelium pellets into microalgal cultures, thereby creating an integrated ecosystem in which filamentous fungi serve both as support matrices for microalgal immobilization and active symbiosis. This mutualism enhances biomass productivity and reduces culture costs.^168^ For example, (Das *et al.*, 2022) demonstrated that co-culture with fungal mycelium significantly increased microalgal biomass yield, lipid accumulation, and wastewater treatment efficiency.^181^ Harvesting the co-culture biomass is greatly simplified, as fungal–algal pellets can be separated efficiently through filtration or simple mechanical methods.^182^ Importantly, fungal co-cultivation increases microalgal tolerance to heavy metals, enhancing the resilience and performance of the treatment system. Furthermore, fungus can protect microalgae from intense light and supply them with nutrients such as minerals and inorganic salts.^181,183^

Crucially, microalgae–fungi consortia have yet to be extensively applied to TWW, but studies on individual organisms show compelling potential. *Chlorella vulgaris* reduced COD and BOD by 95%, removed 95.6% Cr, 89.4% Cu, 93.4% Pb, and 93.8% Zn from tannery effluent.^112^ Fungal isolates such as *A. terreus* demonstrated high chromium adsorption capacity.^184^ Thus, when combined in a consortium, simultaneous removal of COD, nutrients, heavy metals (Cr, Ni, Pb), dyes, and tannins is highly probable, with expected removal efficiencies exceeding 90% across parameters from the results of other industrial effluents. Finally, engineered fungi with enhanced chromium-reducing or pollutant-mineralising genes amplify the consortium's potency. These modified strains offer future pathways for targeted remediation using MFC and scalable biofilm-based reactor designs.

Despite the advantages of biological systems integrated with microalgae, there are considerable challenges to be overcome. Due to the presence of heavy metals and recalcitrant organic compounds, the initial toxicity of the effluents can hinder the development of microalgae. Methods such as chemical pretreatment or adsorption with biochar have been applied to mitigate these impacts and increase the resilience of microalgae. In addition, it is essential to optimize operating conditions, such as light intensity, temperature, and nutrient concentration, to maximize microalgae growth and system efficiency.^103,185^ The incorporation of microalgae into biological processes is an innovative and sustainable approach to purify tannery effluents. These systems not only increase the removal of pollutants but also enable the recovery of high-value biomass. Continuous research and technological advances are essential to overcome today's challenges and expand the use of these systems.

## 7. Future perspectives

One priority lies in deepening the understanding of detoxification mechanisms at the molecular level. It is necessary to move beyond phenomenological exploration and map the transcriptomic and proteomic profiles of robust strains, such as *Chlorella* spp. and *Scenedesmus* spp., under the real stress of tannery effluent. Finding the specific genes responsible for tolerance to chromium speciation, sulfide toxicity, and high salinity will permit the development of more effective bioengineering approaches and the supported selection of microorganisms with greater biological resilience. The change from isolated cultures to the design of microbial consortia represents another interesting advance. Rather than relying on empirical trial-and-error methods, it is worth studying synthetic ecology and metabolic modeling tools to predict synergistic interfaces and inhibit competitive suppression in complex systems. The function of quorum sensing molecules in balancing these communities under heavy metal stress and in the growth of granular biofilms calls for planned verification, to guarantee the long-term functionality of the treatment.

From an engineering standpoint, the main bottleneck today is the scaling of laboratory systems to pilot and industrial units operating in continuous flow. Future research should focus on enhancing important operating parameters, such as hydraulic retention time (HRT) and biomass recirculation rates, in advanced reactor configurations like biofilm photobioreactors (MBBR) or high-rate raceway ponds. The strength of performance under seasonal variabilities and shifts in the load of industrial pollutants is important to legitimize the technical feasibility of these integrated technologies. To consolidate economic viability, it is needed to carry out Life Cycle Assessments (LCA) and Technoeconomic Analyses (TEA) that compare microalgae-based systems with the conventional treatment methods used in the leather industry. These assessments need to weigh not only operating expenses but also the advantages of biomass valorization. Studies mapping the partitioning of heavy metals during thermochemical conversion procedures, such as pyrolysis and hydrothermal liquefaction, are necessary to confirm that by-products like biofuels and biofertilizers fall within biological and environmental safety standards.

Finally, the use of advanced biotechnological tools, such as the genetic engineering of bacteria, fungi and algae to express enhanced chromium-reducing genes, enables targeted remediation. Linking these engineered strains with new low-cost adsorbent materials, such as magnetic biochar or carbon nanostructures, may point to high-efficiency hybrid systems. Such joint efforts will allow phycoremediation to advance from a promising option into a central solution within the circular bioeconomy of the tanning industry.

## 8. Conclusion

Despite the promising results examined in this review, scaling these combined systems to the industrial level still hinges on overcoming key technical bottlenecks and addressing critical scientific gaps. At the molecular level, the gene-regulation networks governing microalgal tolerance to tannery-effluent-specific stressors—particularly chromium speciation, sulphide toxicity, and high salinity—remain poorly characterized. Mapping the transcriptomic and proteomic profiles of promising strains such as *Chlorella* spp. and *Scenedesmus* spp. under real effluent conditions is therefore a priority. Another key challenge lies in the rational design of stable and high-performance consortia. Empirical trial-and-error approaches are unreliable for mitigating competitive exclusion or maintaining functional diversity amid the frequent fluctuations in effluent composition. Advancing toward a predictive design of consortia therefore calls for synthetic ecology and metabolic-modelling tools, together with an evaluation of how quorum-sensing signals can be harnessed to stabilize these microorganisms under heavy-metal stress. From an engineering and economic perspective, the transition from laboratory batch testing to continuous-flow pilot systems represents the most urgent research priority. Several operational questions also remain open, including the optimization of hydraulic detention time (TDH), biomass recirculation rates and the choice of the optimal reactor configuration, whether raceway ponds, tubular photobioreactors or moving bed biofilm (MBBR) reactors, to treat raw effluent (TWW) under fluctuating industrial conditions. To establish realistic cost parameters, there is a crucial need for Life Cycle Assessments (LCA) and Techno-Economic Analyses (TEA) that accurately benchmark these combined systems against conventional treatment routes in the leather industry. In addition, the safe recovery of harvested biomass demands dedicated research, since the accumulation of heavy metals in algal tissues can compromise the viability of by-products such as biofuels, biofertilizers, or pigments. Consequently, mapping and quantifying the partitioning of metals during thermochemical conversion processes, specifically pyrolysis and hydrothermal liquefaction (HTL), together with the establishment of strict biosafety limits, will be essential steps to consolidate the link between wastewater treatment and the objectives of a circular bioeconomy.

## Author contributions

Ricky Rajamanickam: original draft preparation, methodology, supervision, conceptualisation, review & editing, and visualisation. Roshna Parveen: original draft preparation, writing – review and editing, and visualisation. Pollyanna Vanessa dos Santos Lin: original draft preparation; writing – review and editing. Hugo Juarez Vieira Pereira: original draft preparation; writing – review and editing. Dison S. P. Franco: original draft preparation; writing – review and editing. Jordana Georgin: original draft preparation; writing – review and editing. Lucas Meili: methodology, supervision, conceptualisation, writing – review and editing. Rangabhashiyam Selvasembian: methodology, supervision, conceptualisation, writing – review and editing.

## Conflicts of interest

There are no conflicts to declare.

## Data Availability

No primary research results, software or code have been included and no new data were generated or analysed as part of this review.
